# Oleoresins and naturally occurring compounds of *Copaifera* genus as antibacterial and antivirulence agents against periodontal pathogens

**DOI:** 10.1038/s41598-021-84480-7

**Published:** 2021-03-02

**Authors:** Fariza Abrão, Thayná Souza Silva, Claudia L. Moura, Sérgio Ricardo Ambrósio, Rodrigo Cassio Sola Veneziani, Raphael E. F. de Paiva, Jairo Kenupp Bastos, Carlos Henrique Gomes Martins

**Affiliations:** 1 Research Laboratory of Applied Microbiology, University of Franca, Franca, SP Brazil; 2 Nucleus of Research in Sciences and Technology, University de Franca, Franca, SP Brazil; 3 Institute of Chemistry, Department of Fundamental Chemistry, University of São Paulo, São Carlos, SP Brazil; 4 School of Pharmaceutical Sciences of Ribeirão Preto, University of São Paulo, Ribeirão Prêto, SP Brazil; 5 Laboratory of Antimicrobial Testing, Institute of Biomedical Sciences – ICBIM, Federal University of Uberlândia, Av. Pará, 1720, Uberlândia, MG 38405-320 Brazil

**Keywords:** Drug discovery, Microbiology, Molecular biology

## Abstract

Invasion of periodontal tissues by *Porphyromonas gingivalis* and *Aggregatibacter actinomycetemcomitans* can be associated with aggressive forms of periodontitis. Oleoresins from different copaifera species and their compounds display various pharmacological properties. The present study evaluates the antibacterial and antivirulence activity of oleoresins obtained from different copaifera species and of ten isolated compounds against two causative agents of periodontitis. The following assays were performed: determination of the minimum inhibitory concentration (MIC), determination of the minimum bactericidal concentration (MBC), and determination of the antibiofilm activity by inhibition of biofilm formation and biofilm eradication tests. The antivirulence activity was assessed by hemagglutination, *P. gingivalis* Arg-X and Lis-X cysteine protease inhibition assay, and *A. actinomycetemcomitans* leukotoxin inhibition assay. The MIC and MBC of the oleoresins and isolated compounds **1**, **2**, and **3** ranged from 1.59 to 50 μg/mL against *P. gingivalis* (ATCC 33277) and clinical isolates and from 6.25 to 400 μg/mL against *A. actinomycetemcomitans* (ATCC 43717) and clinical isolates. About the antibiofilm activity, the oleoresins and isolated compounds **1**, **2**, and **3** inhibited biofilm formation by at least 50% and eradicated pre-formed *P. gingivalis* and *A. actinomycetemcomitans* biofilms in the monospecies and multispecies modes. A promising activity concerning cysteine protease and leucotoxin inhibition was also evident. In addition, molecular docking analysis was performed. The investigated oleoresins and their compounds may play an important role in the search for novel sources of agents that can act against periodontal pathogens.

Periodontitis is a polymicrobial infection originating from excessive pathogenic biofilm accumulation at the gingival margin, that leads to inflammation in tooth-supporting tissues (i.e., the periodontium)^1^. As periodontitis develops, plaque previously dominated by aerobic species transitions to plaque where strict and facultative anaerobic species such as *Porphyromonas gingivalis* and *Aggregatibacter actinomycetemcomitans* prevail^2,3^.

*P. gingivalis* is an important Gram-negative anaerobic bacteria and stands out as a key pathogen in chronic periodontitis: this species plays a disproportionately important role in depressing and deregulating local immune responses, which culminates in increased virulence of the entire community and causes periodontitis dysbiosis^4^. This bacterium is known to produce a repertoire of virulence factors that could penetrate the gingivae and destroy the tissue directly or indirectly by inducing inflammation^5,6^. The virulence factors of this bacterium include fimbriae, capsules, lipopolysaccharide (LPS), lipoteichoic acids, hemagglutinins, gingipains, outer membrane proteins, and outer membrane vesicles^6–8^.

*A. actinomycetemcomitans* is a Gram-negative bacterium, facultative anaerobe that is associated with the etiology of aggressive periodontitis. This bacterium is also associated with non-oral infections, such as endocarditis, and is a candidate bacterial trigger of anti-citrulline autoimmunity in rheumatoid arthritis^9–11^. Some virulence factors of leukotoxin (*ltx*) are related to evasion of host defense and destruction of the host’s tissues^12^.

The *A. actinomycetemcomitans* virulence potential varies among strains, and specific serotypes/clonal types of this bacterium predominate in individuals with aggressive forms of periodontal disease^13^. *LtxA* is a large pore-forming toxin that belongs to the family of bacterial proteins RTX (Repeats-in-toxin). *LtxA* expression varies widely in vitro although all *A. actinomycetemcomitans* strains have a complete *ltxA* operon. *LtxA* expression has not been fully characterized, but environmental and genetic factors regulate its expression^14–16^.

The main goal of non-surgical periodontal therapy is to control microbial periodontal infection by removing bacterial biofilm, calculus, and toxins from periodontally involved root surfaces^17^. Facilitating antibiotic diffusion into biofilms requires agents that can penetrate and destroy the components of the biofilm matrix and kill bacteria^18^.

Over the last decades, phytodrugs have assumed a prominent part as possible alternative therapy in dentistry^19^. Researchers have been interested in plants with antimicrobial properties and bactericidal potential. Plants belonging to the genus Copaifera L. (Fabaceae-Caesalpinioideae), popularly known as ‘copaiba’ in Brazil, are native to the tropical regions of Latin America and Western Africa. Copaifera oleoresin obtained from the trunk of these trees has become prominent in Brazilian Natural Medicine^20^.

Oleoresin is a product of the secondary metabolism of plants, and it defends the plant against animals, fungi, and bacteria. Oleoresins contain mainly diterpenes, including kaurenoic acid, kaurenol, copalic acid, agathic acid, hardwickiic acid, polyalthic acid, and the sesquiterpenes *β*-caryophyllene, karyophylene oxide, *α*-copaene, *α*-humulene, and *β*-bisabolol, among other compounds^21^. In this sense, our research group has been devoted to investigating *Copaifera* spp.^2,22–31^.

Several studies have highlighted the promising potential of *Copaifera* spp. against bacteria that cause endodontic infections and caries^2,24–26,29^. Therefore, here we aimed to evaluate the antibacterial and antivirulence activity of the oleoresins obtained from *C. paupera*, *C. pubiflora*, and *C. reticulata* and isolated compounds against *P. gingivalis* and *A. actinomycetemcomitans*, which are important pathogens in the development of periodontitis, as well investigation of the interactions between the compounds and bacteria through molecular docking analysis.

## Material and methods

### Plant material and isolated compounds

**Figure 1:**
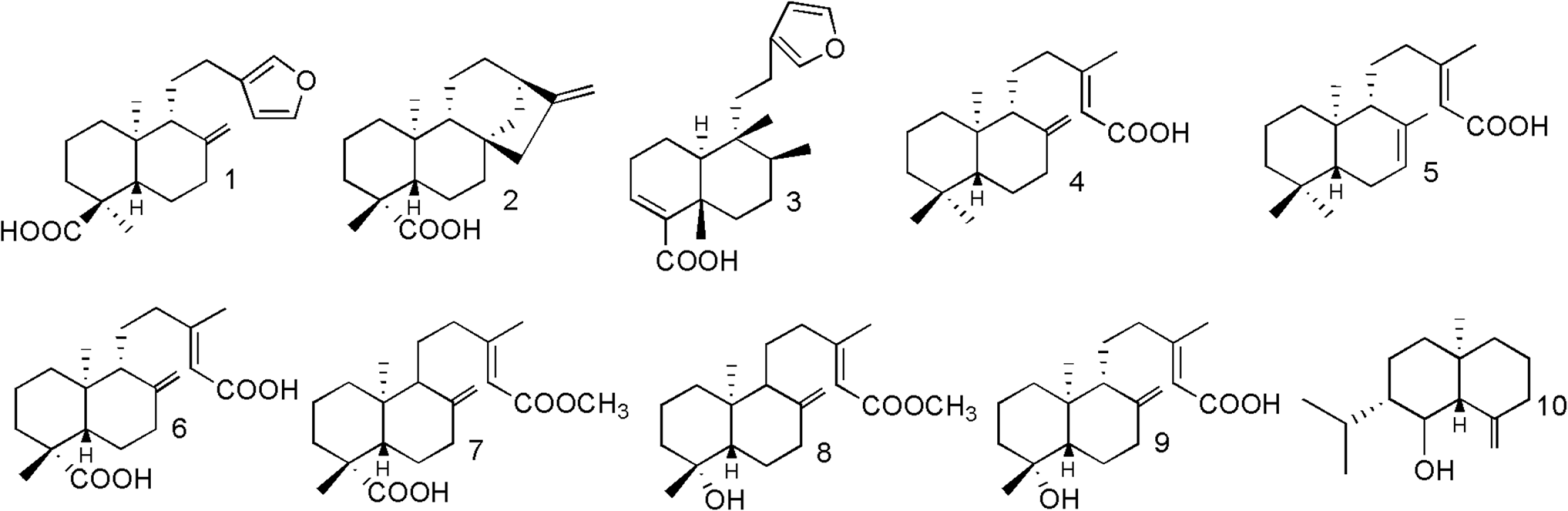
Chemical structures of the tested compounds: polyalthic acid (**1**), kaurenoic acid (**2**), hardwickiic acid (**3**), *ent*-Copalic acid (**4**) and its isomer [(13E)-*ent*-labda-7,13-dien-15-oic acid; **5**], *ent*-agathic acid (**6**), *ent*-agathic acid 15-methyl ester (**7**), *ent*-agatholic acid 15-methyl ester (**8**), *ent*-agatholic acid (**9**), and junenol (**10**).

Authentic *Copaifera* oleoresins were collected in different Brazilian States between 2011 and 2014, and voucher specimens were deposited in the Herbarium of the Brazilian Agricultural Research Corporation (Embrapa Eastern Amazon) and identified as *Copaifera paupera* (Herzog) Dwyer (Xapuri, state of Acre, NID 10/2014), *C. pubiflora* Benth (Mucajaí, state of Roraima, NID 15/2014), and *C. reticulata* Ducke (Brasil Novo, state of Pará, NID 03/2013) by Silvane Tavares Rodrigues^30^. The isolated compounds (Fig. 1) polyalthic acid (**1**), kaurenoic acid (**2**), hardwickiic acid (**3**), *ent*-copalic acid (**4**) and its isomer [(13E)-*ent*-labda-7,13-dien-15-oic acid] (**5**), *ent*-agathic acid (**6**), *ent*-agathic acid 15-methyl ester (**7**), *ent*-agatholic acid 15-methyl ester (**8**), *ent*-agatholic acid (**9**), and junenol (**10**) were isolated from the oleoresins obtained from *Copaifera* spp. and identified by our research group as described previously^22,23,30,32–34^.

### Bacterial strains and antimicrobial assays

The periodontal disease-causing bacteria that were used in this study included standard strains and clinical isolates, namely *P. gingivalis* ATCC 33277 and clinical isolate and *A. actinomycetemcomitans* ATCC 43717 and clinical isolate. The standard strains were obtained from the American Type Culture Collection (ATCC). These bacteria were kept in the UNIFRAN culture collection under cryopreservation at − 80 °C.

The research participants (fifteen subjects) were selected from patients that came to the Dental School, University of Franca, UNIFRAN, Franca, SP, Brazil for periodontic treatment. After the periodontal pockets were evaluated, collections were carried out for a maximum of 5 min, to ensure that the sample was stable. One sample was collected per patient, and the most productive pouch was chosen (> 5 mm deep).

The inclusion criteria were as follows: patients aged over 18 years, of both genders, who had indication for periodontal treatment with over 5-mm deep gingival pockets in at least one of the four facets of the analyzed tooth, and who were examined a dental professional. The patient needed to have at least four teeth.

The exclusion criteria were patients with a history of systemic disease such as rheumatic fever, coronary heart disease, and respiratory diseases; patients that had received systemic or local antibiotic therapy in the six previous months; patients that had used antibiotics or anticoagulants; and patients that had undergone periodontal treatment or dental prophylaxis in the previous six months.

The clinical isolates were collected from patients after approval of the Research Ethics Committee of the University of Franca—CAAE 41530915.9.0000.5495, in accordance with international protection guidelines and the Helsinki Declaration. All the patients signed an informed consent for their participation in this research.

The *P. gingivalis* and *A. actinomycetemcomitans* species were isolated according to the methodology of Esfahani et al.^35^. The species were obtained from patients with chronic and aggressive periodontitis. Ten strains were selected from the total number of isolated and identified bacteria, namely five *P. gingivalis* strains and five *A. actinomycetemcomitans* strains. The Polymerase Chain Reaction (PCR) technique was used to detect the *P. gingivalis* 16SrDNA and *A. actinomycetemcomitans* 16SrDNA genes. DNA was extracted from the strains by employing the Pure Link Microbiome DNA purification kit (Invitrogen, Carlsbad, California, USA); the manufacturer's instructions were followed. The PCR technique was conducted as described by Wu et al*.*^36^.

The Minimum Inhibitory Concentration (MIC; the lowest concentration of the test compound that can inhibit microorganism growth) and the Minimum Bactericidal Concentration (MBC; the lowest concentration of the test compound at which no bacterial growth occurs) were determined as described by Abrão et al.^29^.

The antibiofilm activity was investigated by using two distinct methodologies, in two modes, monospecies and multispecies biofilm. In the first methodology, the Minimum Inhibitory Concentration of Biofilm (MICB_50_) of the most promising metabolites against the bacteria evaluated in this study was determined according to the methodology described by Abrão et al.^29^, on the basis of the minimum concentration of antimicrobial agent that inhibited biofilm formation by at least 50%. In the second methodology, the Minimum Concentration of Biofilm Eradication (MCBE), defined as the lowest concentration that reduced the number of biofilm cells by at least 99.9%, was determined as described by Souza et al.^31^.

### Antivirulence assays

The oleoresins and isolated compounds were used to inhibit virulence factors. The antivirulence assays included assessment of the *P. gingivalis* and *A. actinomycetemcomitans* hemagglutination activity, *P. gingivalis* gingipaine inhibition, and *A. actinomycetemcomitans* leukotoxin inhibition.

### Hemagglutination assay

To perform the hemagglutination assay, which allowed fimbriae to be detected, the methodology of Kikuchi et al.^37^ was applied. *P. gingivalis* and *A. actinomycetemcomitans* colonies that had been grown for five days and 24 h, respectively, were suspended in PBS buffer (Sigma) pH 7.4 and adjusted to an absorbance of 2.0 at 660 nm. The oleoresins and isolated compounds were tested at subinhibitory concentrations (½ MIC). To this end, 150 μL of an oleoresin or isolated compound was mixed with 150 μL of the bacterial suspension, which was followed by incubation at appropriate temperature and atmosphere for each strain (anaerobic chamber at 36 °C for *P. gingivalis* strains and CO_2_ chamber at 36 °C for *A. actinomycetemcomitans* strains) in sterile Eppendorf tubes. After incubation, the bacteria were centrifuged at 6000× g for 5 min, resuspended with 500 μL of PBS buffer (Sigma) pH 7.4, and diluted to 1/32 in 96-well plates. A 50-μL aliquot of each dilution was homogenized with 50 μL of a 2% fresh red blood cell suspension in PBS (Sigma) pH 7.4 and incubated in appropriate atmosphere for 3 h. Hemagglutination was visually assessed after incubation.

### Gingipain inhibition assay

The activity of lysine (Lys-X encoded by the *Kgp* gene) and arginine (Arg-X, encoded by the *RgpA* and *RgpB* genes) gingipaines was determined by using N-(p-tosyl)-Gly-Pro-Lys 4-nitroanilide acetate synthetic substrate salt (Sigma-Aldrich) and Nα-benzoyl-L-arginine-7-amido-4-methyl-coumarin hydrochloride (Sigma-Aldrich), respectively, according to the methodology described by Fujise et al.^38^ and Chen et al.^39^, respectively.

The *P. gingivalis* strains were grown in Brucella broth in anaerobic chamber at 36 °C for 72 h. Then, the bacteria were centrifuged at 6000× g for 5 min and resuspended with PBS (Sigma) pH 7.4 buffer containing 1 mM L-cysteine, to adjust the absorbance at 660 nm to 0.26, and the resulting suspensions were added to the 96-well microplate. The oleoresins and isolated compounds were tested against the *P. gingivalis* strains at subinhibitory concentrations (½ MIC).

The Arg-X activity was assayed in 100 μL of PBS containing 1 mM L-cysteine, 100 μL of substrate, and 5 μL of bacterial suspension. The Lis-X activity was tested in 100 μL of PBS containing 1 mM L-cysteine, 100 μL of substrate, and 50 μL of bacterial suspension. The 7-starch-4-methylcoumarin that was released due to cleavage of the substrates was measured on a fluorimeter (Ascent FL, Thermoscientific, Waltham, MA, USA) upon excitation at 365 nm and emission at 460 nm. The proteolytic activity was compared to the untreated control^40^.

### Leukotoxin inhibition assay

This test was carried out by following the methodology described by de Lima et al.^41^. Mononuclear human leukocytes (LMNS) were isolated from an enriched fraction of venous blood leukocytes by using Histopaque 1077 (Sigma-Aldrich); the manufacturer's instructions were followed. The LMNS-containing fraction was collected, and the cells were washed three times with PBS (1000 rpm for 5 min). The cell pellet was resuspended to a concentration of 5 × 10^6^ cells/mL in RPMI culture medium containing L-glutamine, 10% fetal bovine serum, and penicillin–streptomycin (Sigma-Aldrich). The leukocyte fraction was mixed with an oleoresin or isolated compound at a ratio of 10^6^ cells to 100 μL of oleoresin or isolated compound in sterile tubes. Triton X100 at 0.1% was the positive control. The *A. actinomycetemcomitans* strains were cultivated in TSA broth in a CO_2_ chamber at 36 °C for 24 h. After incubation, the strains were centrifuged at 9000 rpm and 4 °C for 15 min, and the pellet was sonicated (16 kHz at 200 W) to obtain the proteins. After sonication, the supernatant was separated into a sterile tube, and the amount of protein was dosed with the Bicinchoninic Acid Kit for Protein Determination, Sigma-Aldrich). An amount of 500 μg/mL protein was added to sterile tubes containing leukocyte and an oleoresin or isolated compound and incubated in the CO_2_ chamber at 37 °C for 1 h. After incubation, the previously prepared trypan blue solution was added to a concentration of 1.6 mg/mL and kept in the CO_2_ chamber for seven minutes. After that, the dead cells were counted with the aid of the Neubauer chamber, and the results were graphically expressed.

### Molecular docking

The interaction of the isolated compounds polyalthic acid, kaurenoic acid, and hardwickiic acid with the active sites of the enzymes **A**. Arg-X (PDB ID 1CVR) and **B**. Lis-X gingipain (PDB ID 6I9A) from *P. gingivalis* was evaluated my molecular docking. The structure of Lis-X was downloaded in .pdb format and imported into Discovery Studio. The structure was prepared using pH 7.4 for assigning the protonation states of polar hydrogens. The prepared protein was then imported into GOLD, and the active site was defined based on the position of the co-crystallized ligand and on the annotation of active site 5 as found on the .pdb file (xyz coordinated 3.946969 -15.587595 8.143534, 20 Å). The structure of Arg-X handled similarly and prepared with GOLD^42,43^. The active site 1 was used, based on the position of the co-crystallized ligand and assignment on the .pdb file (xyz coordinates 57.619488 23.562437 54.075112, 10 Å). 3D structures of the ligands were downloaded from PubChem and further charge states were generated with Discovery Studio, covering pHs 6.5 to 8.5. Molecular docking was run on GOLD^42,43^ (genetic algorithm approach set to most accurate) and the GoldScore fitness function was used for ranking the poses. 3D Ligand interaction diagrams (LIDs) were prepared with Maestro Release 2020-2, Schrödinger, LLC, New York, NY, 2020. Final illustrations were prepared with the open-source distribution of Pymol, version 2.4.0b0.

### Statistical analysis

Statistical analysis of the data obtained from the gingipain inhibition assay and leukotoxin inhibition assay was accomplished by One Way ANOVA analysis and Tukey's test with the aid of the software GraphPad Prism version 5.00.

## Results and discussion

### MIC and MBC

We used strains that were isolated from patients diagnosed with chronic and aggressive periodontitis. More specifically, we detected five *P. gingivalis* strains and five *A. actinomycetemcomitans* strains and named them PG01 to PG05 and AA01 to AA05, respectively. PCR with the 16srDNA gene helped to confirm the strains. We evaluated the *C. paupera*, *C. pubiflora*, and *C. reticulata* oleoresins and isolated compounds against the *P. gingivalis* and *A. actinomycetemcomitans* strains isolated from patients with periodontitis and against standard strains. Table 1 summarizes the results of these assays.

**Table 1:** Minimum inhibitory concentration and minimum bactericidal concentration of oleoresins and isolated compounds against *P. gingivalis* and *A. actinomycetemcomitans* strains.

Bacteria		Minimum Inhibitory Concentration / Minimum Bactericidal Concentration (µg/mL)													
		*C. reticulata* oleoresin	*C. paupera* oleoresin	*C. pubiflora* oleoresin	**1**	**2**	**3**	**4**	**5**	**6**	**7**	**8**	**9**	**10**	Metronidazole
Clinical isolates	PG 01	6.25/6.25	12.5/12.5	6.25/6.25	6.25/6.25	6.25/6.25	12.5/25	*	*	*	*	*	*	*	–
	PG 02	6.25/6.25	12.5/12.5	12.5/12.5	12.5/12.5	6.25/6.25	12.5/25	*	*	*	*	*	*	*	–
	PG 03	3.12/3.12	3.12/3.12	3.12/3.12	3.12/3.12	6.25/6.25	1.59/1.59	*	*	*	*	*	*	*	–
	PG 04	12.5/12.5	6.25/6.25	12.5/12.5	12.5/12.5	6.25/6.25	12.5/12.5	*	*	*	*	*	*	*	–
	PG 05	6.25/6.25	12.5/12.5	12.5/12.5	12.5/12.5	6.25/6.25	12.5/12.5	*	*	*	*	*	*	*	–
ATCC	ATCC 33277	6.25/12.5	50/50	25/50	6.25/6.25	6.25/6.25	3.12/3.12	*	*	*	*	*	*	*	–
Technique control**	*B. thetaiotaomicron* ATCC 29741	–	–	–	–	–	–	–	–	–	–	–	–	–	2.95/2.95
	*B. fragilis* ATCC 25285	–	–	–	–	–	–	–	–	–	–	–	–	–	1.47/1.47
Clinical isolates	AA 01	100/100	50/50	12.5/12.5	12.5/12.5	400/400	12.5/12.5	*	*	*	*	*	*	*	–
	AA 02	25/25	25/25	6.25/6.25	6.25/6.25	25/50	12.5/25	*	*	*	*	*	*	*	–
	AA 03	100/100	100/100	12.5/25	25/50	400/400	6.25/6.25	*	*	*	*	*	*	*	–
	AA 04	100/100	50/100	6.25/6.25	25/25	400/400	6.25/12.5	*	*	*	*	*	*	*	–
	AA 05	50/50	50/50	12.5/25	25/50	400/400	25/25	*	*	*	*	*	*	*	–
ATCC	ATCC 43717	25/50	12.5/25	25/50	25/25	100/100	25/25	*	*	*	*	*	*	*	–

*PG Porphyromonas gingivalis, AA Aggregatibacter actinomycetemcomitans.*

**1**. polyalthic acid; **2**. kaurenoic acid; **3**. hardwickiic acid; **4**. *ent*-Copalic acid; **5**. isomer [(13E)-*ent*-labda-7,13-dien-15-oic acid]; **6**. *ent*-agathic acid; **7.** *ent*-agathic acid 15-methyl ester; **8**. *ent*- agatholic acid 15-methyl ester; **9**. *ent*-agatholic acid; **10**. Junenol.

*Concentrations considered inactive (> 400 µg/mL).–not tested.

**Technique control strains: *Bacteroides fragilis* (ATCC 25285) and *Bacteroides thetaiotaomicron* (ATCC 29741).

Regarding the tested *P. gingivalis* strains, the MIC and MBC values of the *C. reticulata* oleoresin ranged from 3.12 to 12.5 μg/mL. The MBC results revealed a bactericidal effect against all the strains. The exception was *P. gingivalis* ATCC 33277, against which the *C. reticulata* oleoresin exerted a bacteriostatic action. The *C. paupera* oleoresin provided MIC and MBC values between 3.12 and 50 μg/mL and had a bactericidal effect against all the strains. The *C. pubiflora* oleoresin exhibited MIC and MBC values between 3.12 and 50 μg/mL and was bacteriostatic only against *P. gingivalis* (ATCC 33277). Polyalthic acid (**1**) afforded MIC and MBC values between 3.12 and 12.5 μg/mL and displayed a bactericidal effect against all the strains. Kaurenoic acid (**2**) provided MIC and MBC values of 6.25 μg/mL. Hardwickiic acid (**3**) exhibited MIC and MBC results ranging from 1.59 to 25 μg/mL and exhibited bactericidal activity against PG03, PG04, PG05, and *P. gingivalis* (ATCC 33277).

Concerning the assayed *A. actinomycetemcomitans* strains, the *C. reticulata* oleoresin afforded MIC and MBC results between 25 and 100 μg/mL and exerted a bacteriostatic effect only against *A. actinomycetemcomitans* (ATCC 43717). The MIC and MBC values of the *C. paupera* oleoresin varied from 12.5 to 100 μg/mL, and this oleoresin had a bacteriostatic effect against AA04 and *A. actinomycetemcomitans* (ATCC 43717). The *C. pubiflora* oleoresin exhibited MIC and MBC results between 6.25 and 50 μg/mL and displayed a bactericidal effect against AA01, AA02, and AA04. This same oleoresin exhibited a bacteriostatic effect against AA03, AA05, and *A. actinomycetemcomitans* (ATCC 43717). Isolated compound **1** afforded MIC and MBC results between 6.25 and 50 μg/mL and had a bacteriostatic effect against AA03 and AA05. Isolated compound **2** provided MIC and MBC values between 25 and 400 μg/mL and exerted a bactericidal effect against all the *A. actinomycetemcomitans* strains except AA02. Isolated compound **3** exhibited MIC and MBC results between 6.25 and 25 μg/mL and presented a bacteriostatic effect against AA02 and AA04.

As for isolated compounds **4**–**10**, they afforded MIC values higher than 10 μg/mL against all the tested *P. gingivalis* and *A. actinomycetemcomitans* strains. According to Ríos and Récio^44^ and Gibbons^45^, only crude plant extracts with MIC values lower than 100 μg/mL and isolated substances with MIC values lower than 10 μg/mL are considered as promising sources of antibacterial agents.

Few articles have addressed the antibacterial activity of the oleoresins and isolated compounds investigated in this study. Tincusi et al.^46^ evaluated the antibacterial activity of terpenoids isolated from the *C. paupera* oleoresin against Gram-positive strains. The authors highlighted the results they achieved with copalic acid, polyalthic acid, and kaurenoic acid (MIC lower than 10 μg/mL) against Gram-positive strains and the antibacterial potential of the *C. paupera* oleoresin, used in traditional Peruvian medicine.

In a recent work, Furtado et al.^30^ evaluated the cytotoxic and genotoxic activity of oleoresins obtained from six *Copaifera* species, including *C. reticulata*, *C. paupera*, and *C. pubiflora*. The authors employed the clonogenic efficiency assay to determine the cytotoxicity of the oleoresins extracted from the different species. On the basis of the cytotoxicity results, they selected *C. paupera* and *C. pubiflora* for the genotoxicity assay. Testing with V79 cells (Chinese hamster fibroblasts) revealed that all the oleoresins are cytotoxic: IC_50_ values range from 9.8 to 99.2 μg/mL, but none of the oleoresins is cytotoxic at 2000 mg/kg. Compared to the negative control, V79 cell cultures treated with the *Copaifera* oleoresins do not have significantly different frequency of micronuclei, demonstrating the absence of genotoxic effect. Here, the *C. reticulata*, *C. paupera*, and *C. pubiflora* oleoresins showed cytotoxicity: their IC_50_ values were 60.8, 40.6, and 54.0 μg/mL, respectively. Comparison of these IC_50_ values with the MIC results (from 3.12 to 12.5 μg/mL for *C. reticulata*, from 3.12 to 50 μg/mL for *C. paupera*, and from 3.12 to 25 μg/mL for *C. pubiflora* against the evaluated *P. gingivalis* strains) showed that microorganism inhibition was due to the oleoresin antibacterial activity and not to the cytotoxic action. With respect to the tested *A. actinomycetemcomitans* strains, the MIC values of the oleoresin were higher than the IC_50_ values found by Furtado et al.^30^, but we also attributed microorganism inhibition to the antibacterial activity and not to the cytotoxicity of the oleoresins.

According to Bakri and Douglas^47^, for new therapeutic agents to translate into effective in vivo therapies for periodontitis, they must be active against biofilms rather than just planktonic cells. Alves et al.^48^ stated that antibiofilm compounds act in distinct ways: they can have a preventive effect through inhibition of biofilm formation or a therapeutic effect by acting on already established biofilms. Therefore, we decided to evaluate the two possible antibiofilm activities by conducting biofilm inhibition and eradication assays.

### Antibiofilm assays

**Figure 2:**
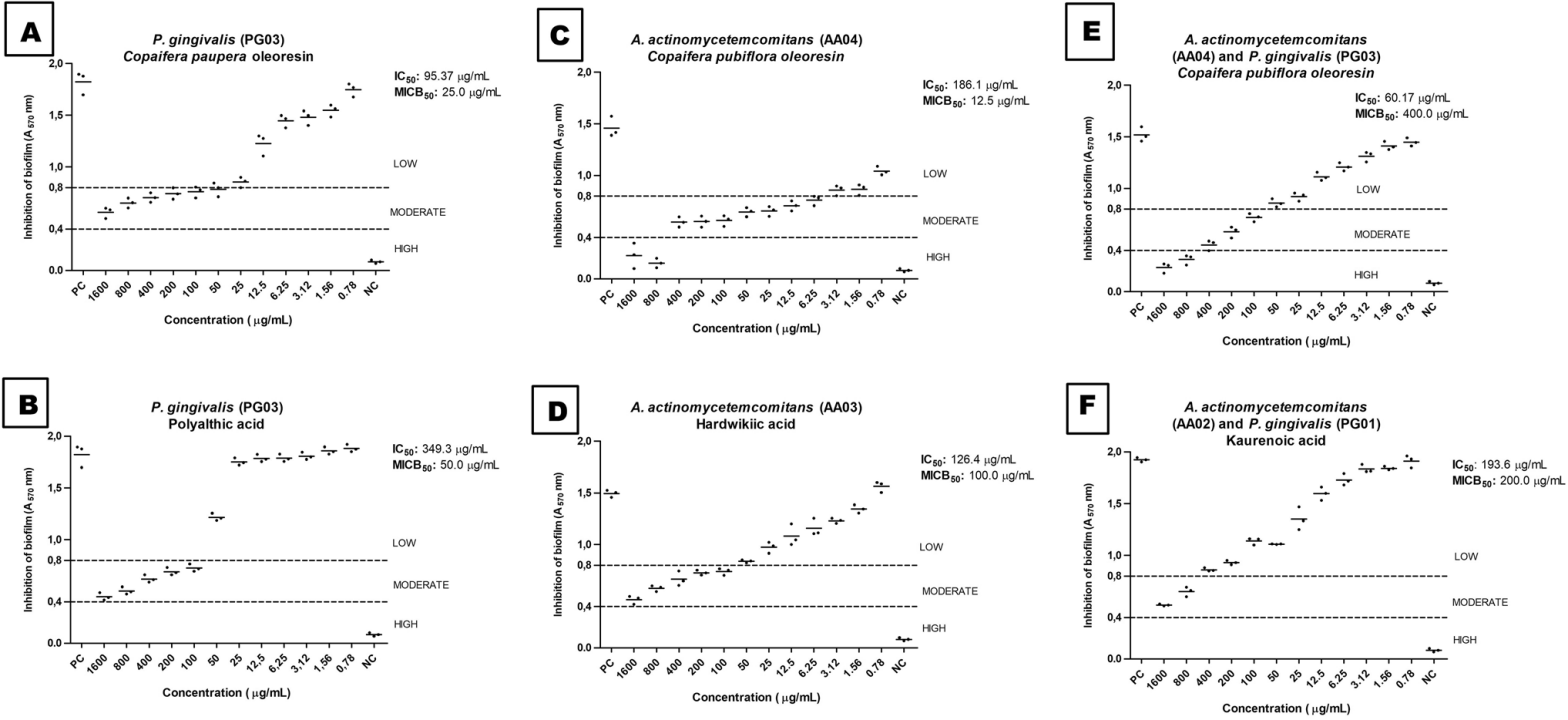
Biofilm inhibition of *A. actinomycetemcomitans* and *P. gingivalis* in the monospecies (**A**,**B**,**C**,**D**) and multispecies (**E**,**F**) modes.

To carry out the biofilm inhibition and eradication tests on the *P. gingivalis* and *A. actinomycetemcomitans* strains, first we had to verify the ability of the analyzed strains to grow in the sessile mode. All the strains formed monospecies and multispecies biofilms. In the antibiofilm activity assay, we evaluated concentrations from 0.78 to 1600 µg/mL (Fig. 2A–F). To determine the anti-biofilm activity of the oleoresins and isolated compounds, we used two techniques: optical density reading (which evaluates the presence of biofilm mass) at 570 nm and microorganism count expressed in log_10_ and colony forming units per milliliter (CFU/mL).

Concerning the inhibitory activity of the oleoresins against the monospecies biofilms, the *C. paupera* oleoresin exhibited IC_50_ and MICB_50_ of 95.37 and 25.0 µg/mL, respectively, against PG03 (Fig. 2A). The *C. pubiflora* oleoresin provided IC_50_ and MICB_50_ of 186.1 and 12.5 µg/mL, respectively, against AA04 (Fig. 2C). In the case of the multispecies mode, the *C. pubiflora* oleoresin at 800 and 1600 µg/mL displayed high antibacterial activity, with IC_50_ of 60.17 µg/mL and MICB_50_ of 400 µg/mL against a combination of AA04 and PG03 (Fig. 2E).

According to Fux et al.^49^, the drug concentration that is required to kill bacteria in the sessile mode may be 10 to 1000 times higher than the concentration that is necessary to kill bacteria in the planktonic mode. Here, the MICB_50_ values of the evaluated oleoresins and isolated compounds against the monospecies mode ranged from 12.5 to 100 μg/mL (Fig. 2A–D). For the tested *P. gingivalis* strains, the MICB_50_ values were 8 to 251 times higher than the MIC values, corroborating the data established by Fux et al.^49^. With respect to the assayed *A. actinomycetemcomitans* strains, the MICB_50_ values were 2 to 16 times higher than the MIC values and are considered promising for anti-biofilm activity.

Souza et al.^31^ evaluated the anti-biofilm activity of *ent*-copalic acid against *Actinomyces naeslundii* and *Peptoestreptococcus anaerobius*, which cause endondontic infections. *ent*-Copalic acid at 500 and 2000 μg/mL inhibits biofilm formation by 50% for *A. naeslundii* and *P. anaerobius*. Herein, isolated compounds **1**, **2**, and **3** presented MICB_50_ of 50, 100, and 200 μg/mL against the monospecies and multispecies biofilms (Fig. 2B,D,F). Therefore, our results were superior to the results of Souza et al.^31^.

Abrão et al.^29^ assessed the anti-biofilm activity of *C. duckei* and its isolated compound polyalthic acid against *P. gingivalis* (ATCC 33277) and found MICB_50_ of 200 μg/mL for the oleoresin and 6.25 μg/mL for polyalthic acid. In our studies, the *C. pubiflora* and *C. paupera* oleoresins exhibited MICB_50_ of 12.5 and 25 μg/mL against the *A. actinomycetemcomitans* and *P. gingivalis* clinical isolates, respectively (Fig. 2A,C). Regarding isolated compound **1**, which exhibited MICB_50_ of 50 μg/mL against PG03 (Fig. 2B), which was lower than the result found in the studies by Abrão et al.^29^. These results confirmed the potential antibiofilm action of the *Copaifera* species oleoresins and their isolated compounds and reaffirmed the selectivity toward more resistant strains in the bacterial biofilm of patients with periodontal pouch^50,51^.

**Figure 3:**
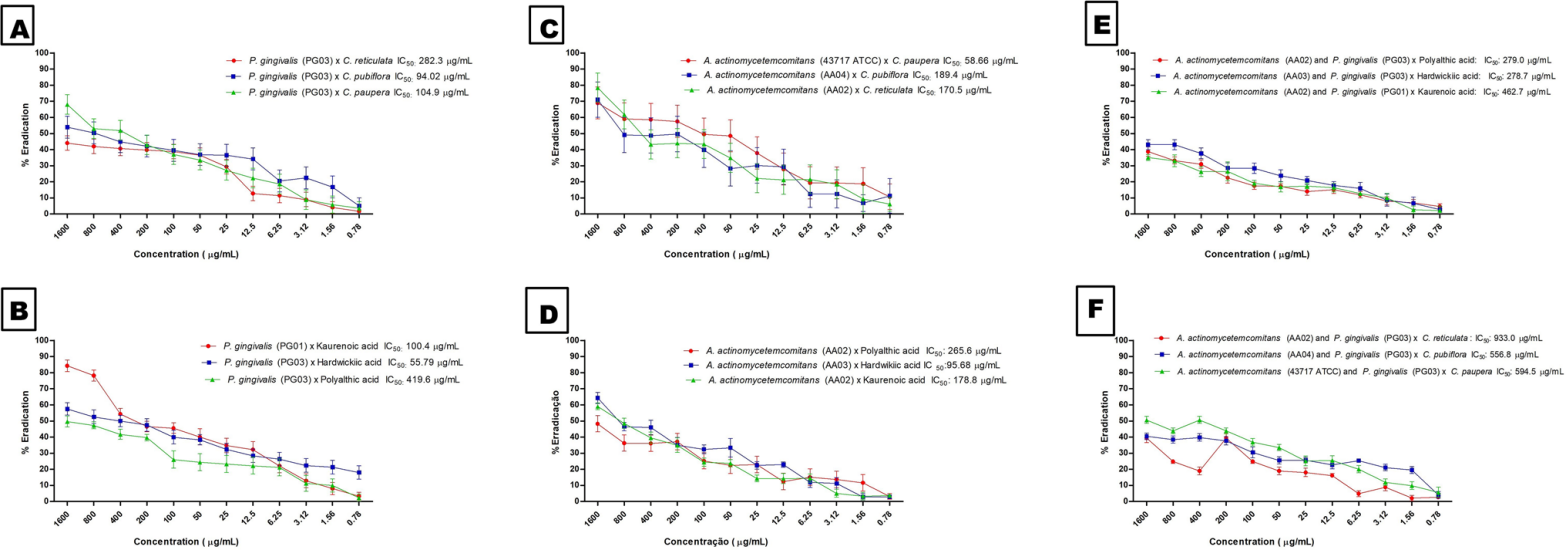
Biofilm eradication of *A. actinomycetemcomitans* and *P. gingivalis* in the monospecies (**A**,**B**,**C**,**D**) and multispecies (**E**,**F**) modes.

Graphs in Fig. 3 illustrate the biofilm eradication activity of the investigated oleoresins and isolated compounds against the tested bacteria in the monospecies and multispecies modes. In the monospecies mode, isolated compound **3** provided the best activity against PG03 (IC_50_ of 55.79 µg/mL) (Fig. 3B), whereas the *C. paupera* oleoresin exhibited the best result against *A. actinomycetemcomitans* (ATCC 43717) (IC_50_ of 58.66 µg/mL) (Fig. 3C). With respect to the multispecies mode, isolated compound **3** eradicated the combination of AA03 and PG03 with IC_50_ of 278.7 µg/mL (Fig. 3E).

Souza et al.^31^ evaluated the biofilm eradication activity of *ent*-copalic acid against *A. naeslundii* and *P. anaerobius* biofilms. These authors calculated the Minimum Inhibitory Concentration of Biofilm Eradication, which is defined as the concentration of the substance that is able to reduce biofilm cells by 99.9%. The *ent*-copalic acid at 1000 and 62.5 μg/mL eradicates *A. naeslundii* biofilm cells and *P. anaerobius* biofilm cells, respectively. We determined the biofilm eradication activity on the basis of IC_50_, which is defined as the concentration of an inhibitor that results in half the inhibition of a response as compared to the control group^52^. In the case of the *P. gingivalis* clinical isolates, the IC_50_ of the evaluated oleoresins and isolated compounds ranged from 94.02 to 282.3 μg/mL and from 55.79 to 419.6 μg/mL, respectively (Fig. 3A,B). For *A. actinomycetemcomitans*, the oleoresins and isolated compounds exhibited IC_50_ from 58.66 to 189.4 μg/mL and from 95.68 to 265.6 μg/mL, respectively (Fig. 3C,D). These results were more promising than the values reported by Souza et al.^31^.

In their natural environments, most biofilms probably consist of a consortium of species that influence each other synergistically or antagonistically. However, there is little knowledge of their structure, characteristics (including community dynamics), and response to antimicrobial agents^53^.While monospecies biofilms have been extensively studied, little is known about multispecies biofilms and their interactions^53^. Interactions between microorganisms are complex and play an important role in the pathogenesis of infections. These interactions may range from fierce competition for nutrients and niches to highly evolved cooperative mechanisms between different species that support their mutual growth^54^.

### Hemagglutination assay

Herein, we evaluated the oleoresins and isolated compounds at sub-inhibitory concentrations. Concerning the *A. actinomycetemcomitans* strains, the oleoresins and isolated compounds inhibited the hemagglutination activity at all the assayed dilutions. Controls were performed by assessing the ability of all the strains to hemagglutinate blood suspension. All the *P. gingivalis* strains were capable of hemagglutination, and the oleoresins and isolated compounds inhibited their hemagglutination activity at all the tested dilutions. In *P. gingivalis*, the hemagglutination capacity is associated with the process of adhesion to gum cells and later lysis of red blood cells for iron capture^55–58^.

*A. actinomycetemcomitans* exhibits many virulence factors (fimbriae, hemagglutinin, capsule, lipopolysaccharide, outer membrane vesicles, and enzymatic activities) that can disrupt host defense mechanisms and initiate tissue destruction; however, no specific invasion mechanism has been identified^59^. For Nakagawa et al.^60^, the *A. actinomycetemcomitans* cellular invasion rate is low as compared to *P. gingivalis*. In the studies by Meyer et al.^61^, invasion of human cells by *A. actinomycetemcomitans* was limited to some bacterial strains, approximately 25% of invasive *A. actinomycetemcomitans*. Löhr et al.^62^ evaluated the ability of polyphenols obtained from *Myrothamnus flabellifolia* to inhibit hemagglutination in the case of *P. gingivalis*, and they found that the polyphenols at 1000, 500, 100, 50, and 1 μg/mL inhibit this activity at all the tested dilutions (1 to 1:64). In the present study, we examined subinhibitory concentrations of the oleoresins and isolated compounds, from 0.79 to 3.12 μg/mL, and verified the promising potential of the *C. paupera*, *C. pubiflora*, and *C. reticulata* oleoresins and their isolated compounds **1**, **2**, and **3** to inhibit the *P. gingivalis* and *A. actinomycetemcomitans* hemagglutination activity.

### Gingipain inhibition assay

**Figure 4:**
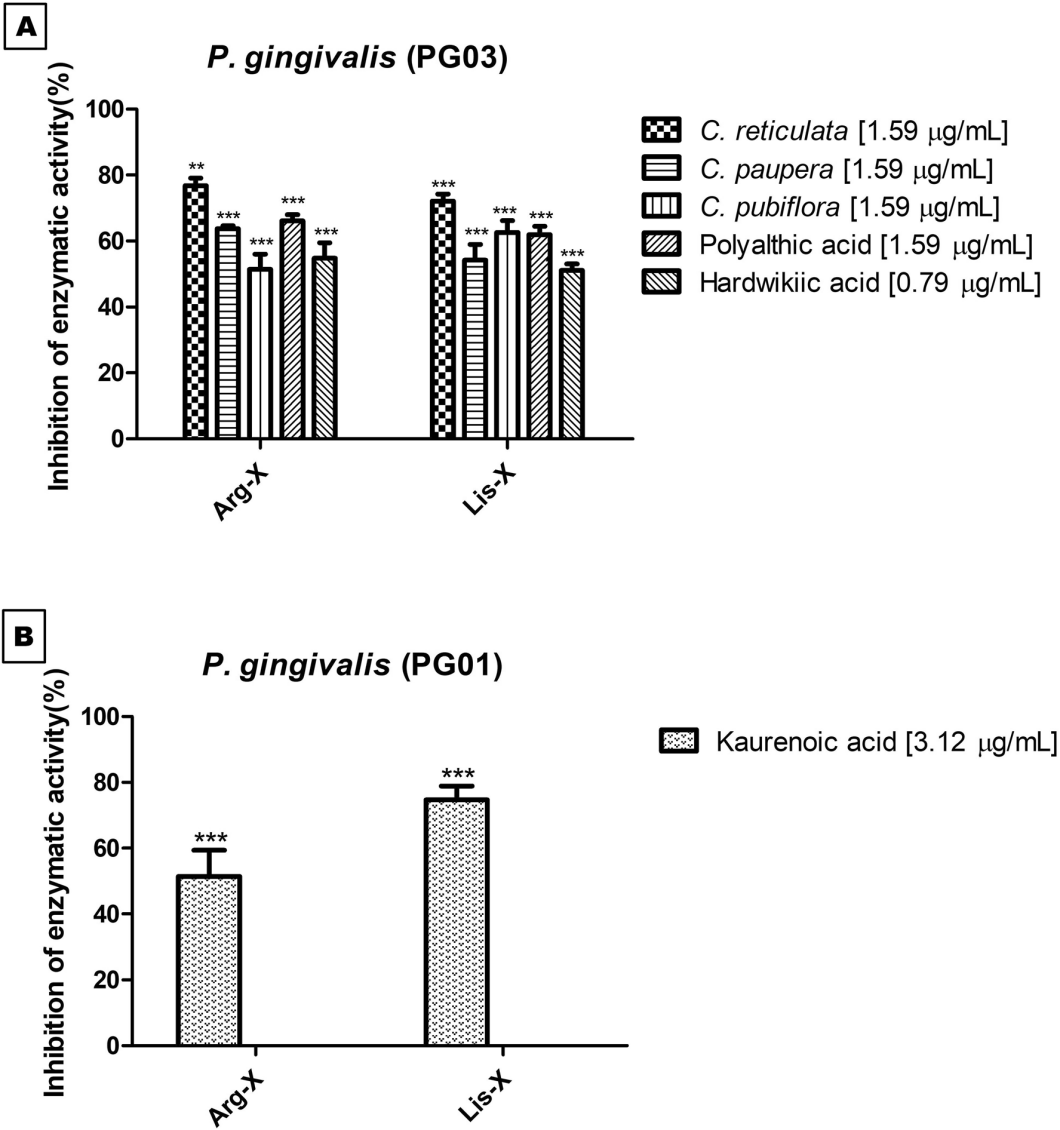
Inhibition of the Arg-X and Lys-X enzymatic activity (%) by the *C. reticulata, C. paupera,* and *C. pubiflora* oleoresins (**A**) and the isolated compounds kaurenoic acid (**B**), polyalthic acid, and hardwickiic acid (**A**). Values are expressed as mean and standard deviation in triplicate. Double asterisks (*P* < 0.01) and triple asterisks (*P* < 0.001) indicate significantly different values versus control values. Data were analyzed using GraphPad Prism software version 5.00 (available https://www.graphpad.com/scientific-software/prism/).

We evaluated the ability of the *C. paupera*, *C. pubiflora*, and *C. reticulata* oleoresins and isolated compounds **1**, **2**, and **3** to inhibit the gingipain activity of the *P. gingivalis* strains PG01 and PG03. The tested concentrations were sub-inhibitory, and the results are shown in Fig. 4A,B.

The *C. reticulata*, *C. paupera*, and *C. pubiflora* oleoresins inhibited the Arg-X enzymatic activity by 78.81 ± 4.02%, 63.71 ± 1.62%, and 51.47 ± 7.86%, respectively, and the Lis-X enzymatic activity by 72.09 ± 3.59%, 54.24 ± 8.11%, and 62.66 ± 6.13%, respectively (Fig. 4A). Isolated compounds **1**, **2**, and **3** inhibited the Arg-X enzymatic activity by 66.11 ± 3.28%, 80.04 ± 2.69%, and 54.78 ± 8.11%, respectively, and the Lis-X enzymatic activity by 61.94 ± 4.38%, 74.66 ± 4.21%, and 51.10 ± 3.29%, respectively (Fig. 4A,B). The proteases produced by *P. gingivalis* play a crucial role in this assacarolytic bacterium: this microorganism does not break down carbohydrates to produce energy, so it needs to degrade host proteins and amino acids^63^. *P. gingivalis* proteases are known as gingipaines and include lysine-specific gingipaines, encoded by the *Kpg* gene, and arginine-specific gingipaines, encoded by the *RgpA* and *RgpB* genes^64^.

Löhr et al.^62^ investigated the ability of *Myrothamnus flabellifolia* Welw polyphenols to inhibit the Arg-X and Lis-X enzymatic activity. After contact with 1 μg/mL polyphenols for 1 min, inhibition is 50%. Prolonged incubation does not increase the anti-protease effects. Polyphenols at 50 and 100 μg/mL inhibit Arg-gingipain by 70–80% and by about 80%, respectively. As for Lys-gingipain, inhibition is only 50%. The authors indicated that *M. flabellifolia* polyphenol is a potent inhibitor of Arg-X activity.

Here, we evaluated the antivirulence activity of the oleoresins obtained from the *Copaifera* species and isolated compounds by the Arg-X and Lis-X enzyme inhibition assay. The oleoresins and isolated compounds inhibited at least 50% of the Arg-X and Lys-X enzymatic activity (Fig. 4).

The structures of Arg-X and Lis-X gingipaines are described as resembling a tooth^65,66^, with a crown encompassing the N-terminal subdomain (NSD) and C-terminal subdomain (CSD), see Supplementary Fig. 1. The catalytic site is part of the CSD. The “root” is composed of a IgSF. The hydrolase activities (Cys-proteinase, EC 3.4.22.47) of Arg-X and Lis-X gingipaines are related to different sets of catalytic residues. For Arg-X, H444 and C477 form the the catalytic dyad, while for the Lis-X enzyme the catalytic triad is composed by H444, C477 and D388, as shown in recent studies^66^.

**Figure 5:**
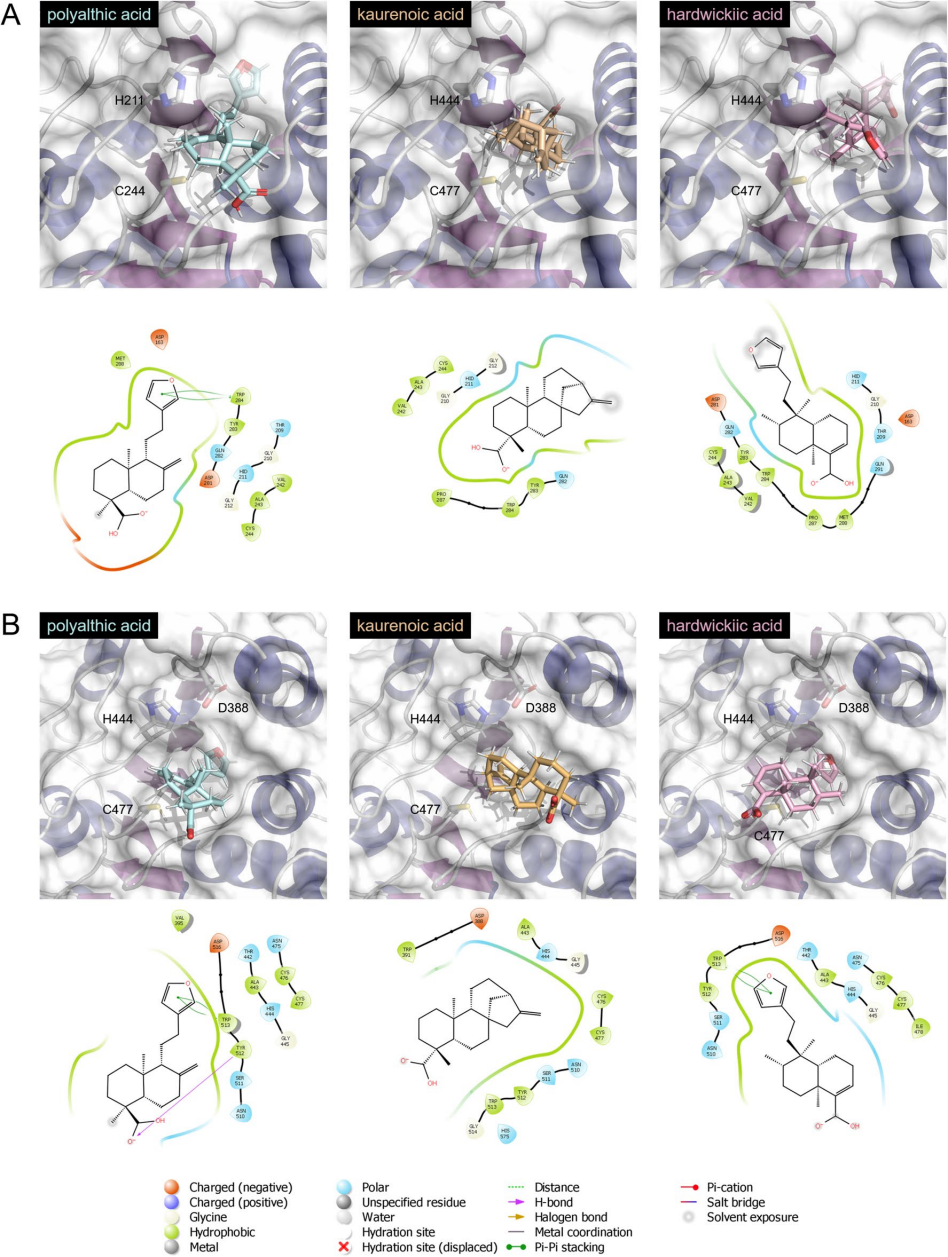
The interaction of the isolated compounds kaurenoic acid, polyalthic acid, and hardwickiic acid with the active sites of the enzymes (**A**). Arg-X (PDB ID 1CVR) and (**B**). Lis-X gingipain (PDB ID 6I9A) from *P. gingivalis* was evaluated my molecular docking using the GOLD^42,43^. The active sites of each enzyme are also shown. For Arg-X, H444 and C477 form the the catalytic dyad, while for the Lis-X enzyme the catalytic triad is composed by H444, C477 and D388, as shown in recent studies^4^. Ligand interaction diagrams (LIDs) shown in details the intermolecular interactions in each case. LIDs were prepared with Maestro Release 2020-2, Schrödinger, LLC, New York, NY, 2020.

To obtain further information on the mechanism of inhibition of Arg-X and Lis-X gingipain, the GOLD molecular docking suite was used^42,43^. The isolated compounds polyalthic acid, kaurenoic acid, and hardwickiic acid were docked into the active sites of the enzymes Arg-X (PDB ID 1CVR) and Lis-X gingipain (PDB ID 6I9A) from *P. gingivalis* (Fig. 5). The results obtained here, in combination with the ligand interaction diagrams, demonstrate that all three free compounds can interact with the active sites of both enzymes. Interestingly, the compounds endowed with the furan-containing flexible “arm” (polyalthic acid and hardwickiic acid) could be expected to bind more tightly with the active site binding pocket. This trend is observed in the GoldScore Fitness scores. For Lys-X, polyalthic acid and hardwickiic generated posed with better interaction with the active site of the enzyme than kaurenoic acid (See Table 2). For Arg-X, on the other hand, the only compound that was predicted to bind tightly with the active site was polyalthic acid (see Table 2).

**Table 2:** GoldScore fitness results obtained for the binding of the isolated compounds polyalthic, kaurenoic acid and ( +)-hardwickiic acid into the active sites of Arg-X and Lys-X gingipaines.

Isolated compound	Arg-X	Lys-X
Polyalthic acid	45.0830	55.9521
Kaurenoic acid	12.8104	32.0326
( +)-hardwickiic acid	6.4601	51.6448

The molecular docking analysis indicates that, although some of the compounds are predicted to have the potential to bind to the active sites of both Arg-X and Lys-X, gingipaines, this factor alone does not account to for inhibitory potencies observed experimentally in vitro*.* It suggests that other forms of inhibition are taking place experimentally. Allosteric inhibition, for example, is not uncommon for proteases^67^.

### Leukotoxin inhibition assay

**Figure 6:**
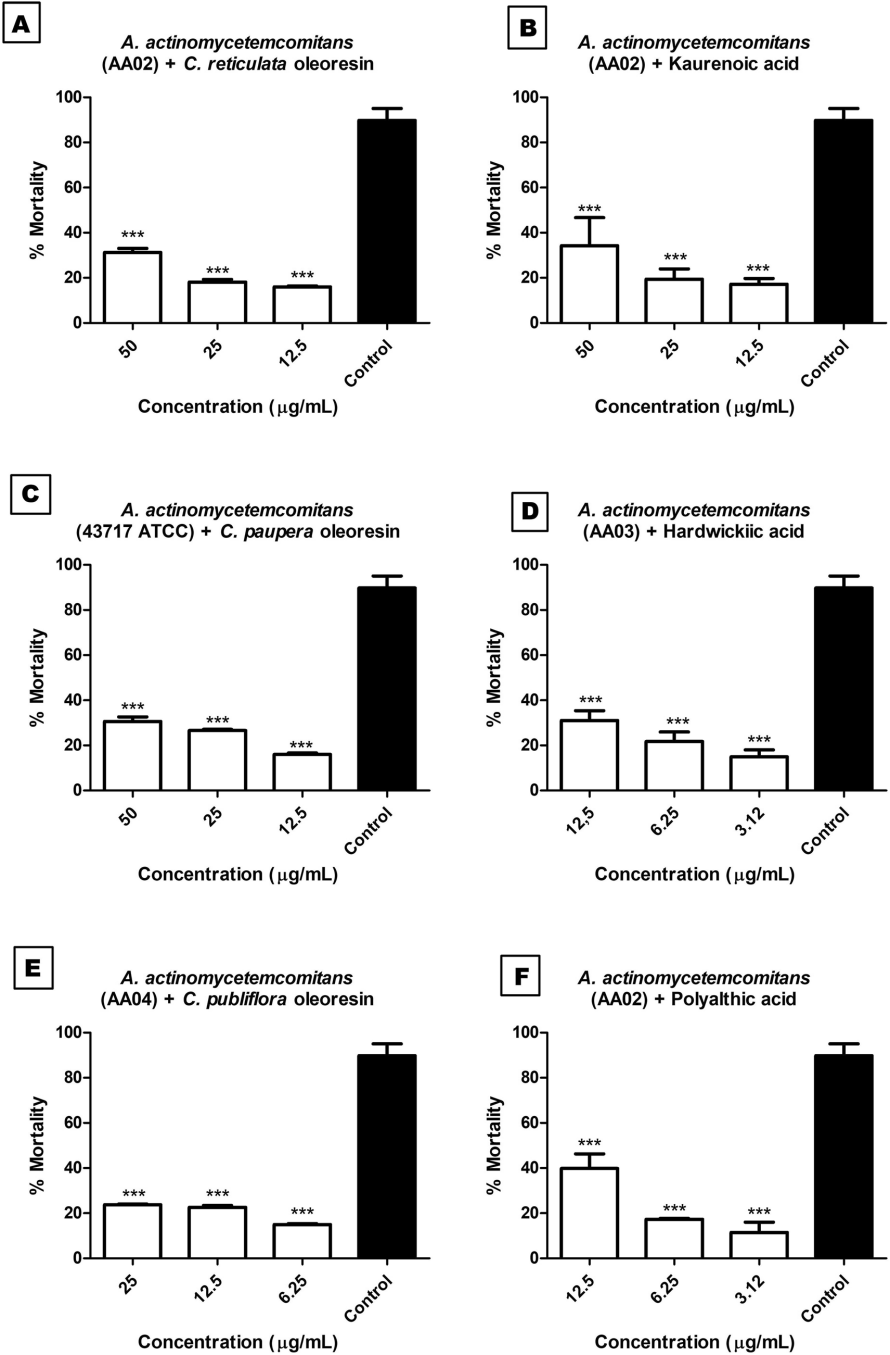
Mortality percentage of human mononuclear cells after exposure to the oleoresins (**A**,**C**,**E**) and the isolated compounds kaurenoic acid (**B**), polyalthic acid (**F**), and hardwickiic acid (**D**) and the control (Triton-X). Values are expressed as mean and standard deviation in triplicate. Triple asterisks (*P* < 0.001) indicate significantly different values versus control values. Data were analyzed using GraphPad Prism software version 5.00 (available https://www.graphpad.com/scientific-software/prism/).

We exposed different concentrations of the *C. paupera*, *C. pubiflora*, and *C. reticulata* oleoresins and isolated compounds **1**, **2**, and **3** (MIC, ½ MIC, and 2 × MIC) to 10^6^ cells/mL of LMNS and 500 μg/mL of *A. actinomycetemcomitans* proteins. The results in Fig. 6A–F showed that the oleoresins and the isolated compounds inhibited *A. actinomycetemcomitans* leucotoxins: they reduced the leukocyte mortality percentage to less than 50%.

The *ltxA* gene encodes leukotoxin; the *ltxB* and *ltxD* genes encode proteins required for toxin secretion; and the *ltxC* gene encodes acyl transferase production, which underlies toxin transformation from protoxin to the active form^51,68^. The presence of leukotoxin has been associated with the *A. actinomycetemcomitans* ability to escape the main line of defense in the periodontal pocket and contributes to the pathogenesis of periodontal disease^51,69^. Leukotoxic activity is determined by a cytolytic action that kills human polymorphonuclear leukocytes, T lymphocytes, and macrophages. In contrast, epithelial and endothelial cells, fibroblasts, and platelets are resistant to this action^51,69,70^.

In the present study, we assessed the *A. actinomycetemcomitans* leukotoxin inhibition by counting the number of viable cells by Trypan Blue staining. This methodology is based on the observation that viable cells are impermeable to the dye, whereas nonviable cells are permeable to Trypan Blue, which enters pores in the cell membrane^71^. Here, exposure of the *A. actinomycetemcomitans* strains to the *C. paupera*, *C. pubiflora*, and *C. reticulata* reduced leukocyte mortality to between 15.93 and 31.19% (Fig. 6A,C,E). Isolated compounds **1**, **2**, and **3** diminished leukocyte mortality to between 11.44 and 39.83% (Fig. 6B,D,F). There are no literature data on the inhibition of *A. actinomycetemcomitans* leukotoxins by compounds isolated from natural products, so we could not compare the efficiency of the oleoresins and isolated compounds evaluated in the present study with literature results. Compared to the control group, the oleoresins and isolated compounds abated the effect of leukotoxins and decreased leukocyte mortality to below 50%.

## Conclusion

The *C. paupera*, *C. pubiflora*, and *C. reticulata* oleoresins and the isolated compounds polyalthic acid, kaurenoic acid, and hardwickiic acid have promising antibacterial activity and monospecies and multispecies antibiofilm activity against major pathogens that cause periodontitis, namely *A. actinomycetemcomitans* and *P. gingivalis*. Promising antivirulence activity was evident in the hemagglutination assays, *P. gingivalis* cysteine protease inhibition (Arg-X and Lys-X) assays, and *A. actinomycetemcomitans* leukotoxin inhibition assays. The mechanism of inhibition of Arg-X and Lys-X by the isolated compounds was further studies by molecular docking. Direct binding of the small molecules to the active site of the gingipaines does not completely explain the potent inhibitory activities of the compounds observed experimentally (> 74% for kaurenoic acid, for example). Therefore, allosteric inhibition is proposed.

## Supplementary Information

Supplementary Information
