# Supplementary material for: Oleoresins and naturally occurring compounds of Copaifera genus as antibacterial and antivirulence agents against periodontal pathogens
Source: Sci Rep. 2021 Mar 2;11:4953. doi: 10.1038/s41598-021-84480-7 (PMC7925542; doi:10.1038/s41598-021-84480-7)
Supplement: Supplementary file 1 — Supplementary Information [file 41598_2021_84480_MOESM1_ESM.pdf]

Oleoresins and naturally occurring compounds of *Copaifera* genus as antibacterial and antivirulence agents against periodontal pathogens

Fariza Abrão[1], Thayná Souza Silva[1], Claudia L. Moura[1], Sérgio Ricardo Ambrósio[2], Rodrigo Cassio Sola Veneziani[2], Raphael E. F. de Paiva[3], Jairo Kenupp Bastos[4], Carlos Henrique Gomes Martins[1,5*]

[1] *Research Laboratory of Applied Microbiology, University of Franca, Franca, SP, Brazil*
[2] *Nucleus of Research in Sciences and Technology, University de Franca, Franca, SP, Brazil*
[3] *Institute of Chemistry, Department of Fundamental Chemistry, University of São Paulo, São Carlos, SP, Brazil*
[4] *School of Pharmaceutical Sciences of Ribeirão Preto, University of São Paulo, Ribeirão Preto, SP, Brazil*
[5] *Laboratory of Antimicrobial Testing, Institute of Biomedical Sciences – ICBIM, Federal University of Uberlândia, Uberlândia, MG, Brazil*

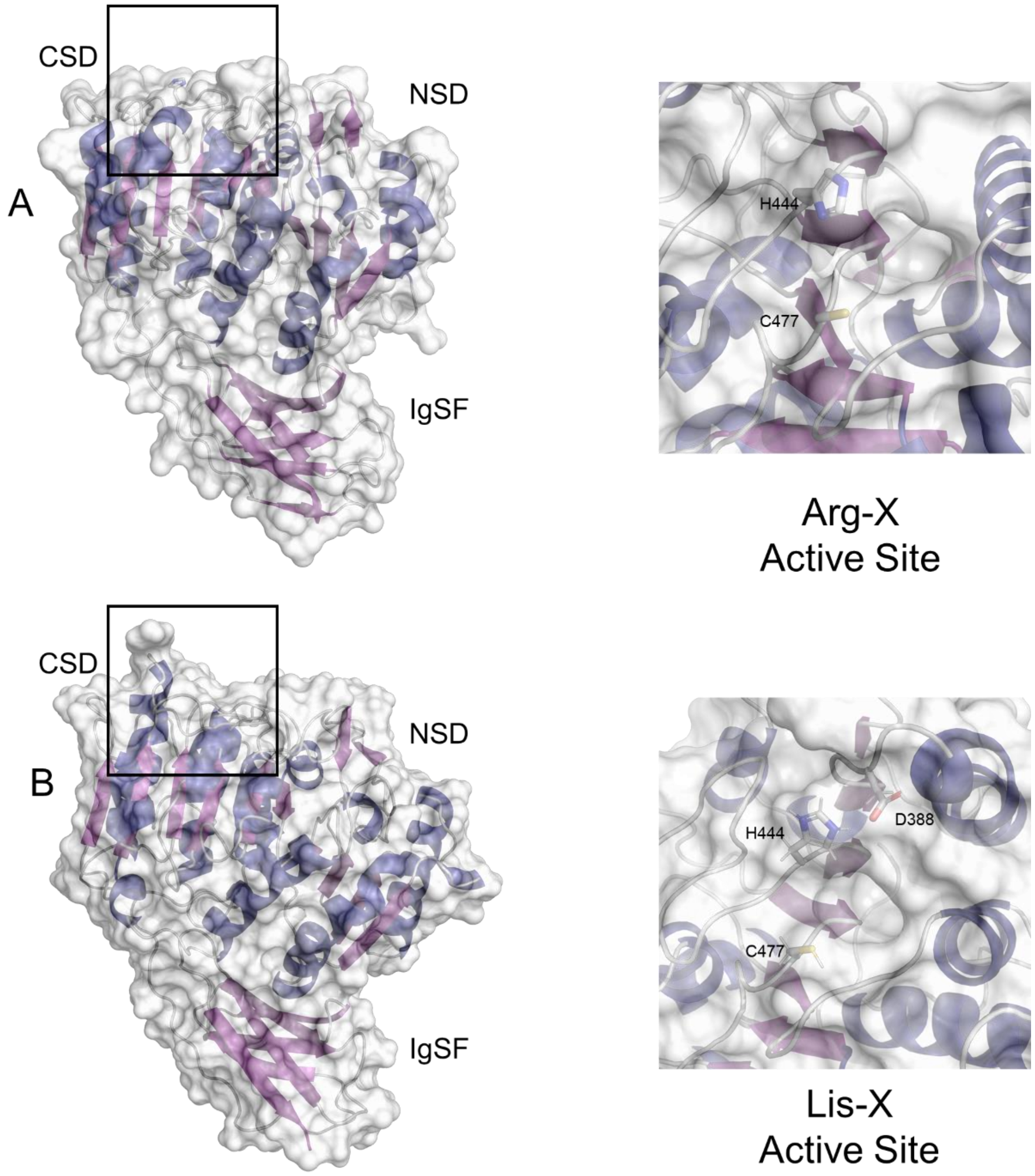

Figure S1. Illustration of the target proteins A. Arg-X (PDB ID 1CVR) and B. Lis-X gingipain (PDB ID 6I9A) from *P. gingivalis*. The active sites of each enzyme are also shown. For Arg-X, H444 and C477 form the the catalytic dyad, while for the Lis-X enzyme the catalytic triad is composed by H444, C477 and D388, as shown in recent studies.[1]

Reference

1. Guevara, T. *et al.* Structural determinants of inhibition of Porphyromonas gingivalis gingipain K by KYT-36, a potent, selective, and bioavailable peptidase inhibitor. *Sci. Rep.* **9**, 4935 (2019).
